# Conditional Latent Diffusion for Controllable Palpebral Conjunctiva Image Generation Toward Hemoglobin Assessment

**DOI:** 10.3390/diagnostics16172715

**Published:** 2026-08-25

**Authors:** Amaal Ibrahim Alhjori, Hajar Mohammedsaleh Alharbi, Nahed Abdulaziz Alowidi

**Affiliations:** Department of Computer Science, King Abdulaziz University, Jeddah 21589, Saudi Arabia; hmsalharbi@kau.edu.sa (H.M.A.); nalowidi@kau.edu.sa (N.A.A.)

**Keywords:** latent diffusion model, palpebral conjunctiva, conditional image generation, medical image generation, synthetic medical images, hemoglobin assessment, diffusion models, generative adversarial networks, cross-population generalizability, computational medical imaging

## Abstract

**Background/Objectives:** Deep learning-based medical imaging applications often require large and diverse datasets to achieve reliable performance. However, publicly available palpebral conjunctiva datasets for non-invasive hemoglobin assessment remain limited in both size and demographic diversity. The objective of this study was to develop and evaluate a conditioning-guided Latent Diffusion Model (LDM) for controllable palpebral conjunctiva image synthesis under limited-data conditions. **Methods:** The proposed framework employs an image-to-image latent diffusion strategy conditioned on continuous hemoglobin values together with gender and country information to generate realistic synthetic conjunctiva images with controllable clinical and demographic characteristics. The proposed LDM was compared with GAN-based approaches, including cDCGAN and StyleGAN2-ADA, using evaluation criteria covering image realism, diversity, conditioning consistency, and computational efficiency. Analyses of frequency-domain characteristics, zero-shot cross-population evaluation, and blinded clinical assessment were performed for the proposed LDM. **Results:** The proposed LDM achieved the lowest FID score (15.86±0.23) among the evaluated models, indicating superior image realism relative to cDCGAN and StyleGAN2-ADA, while maintaining image diversity comparable to StyleGAN2-ADA and substantially outperforming cDCGAN. Conditioning evaluation demonstrated strong consistency between the target hemoglobin values and the generated images, achieving a Pearson correlation coefficient of r=0.910±0.025. Frequency-domain analysis, zero-shot cross-population evaluation, and blinded clinical assessment further supported the realism, structural consistency, and clinical plausibility of the generated images. **Conclusions:** The findings demonstrate the potential of conditional latent diffusion models for controllable palpebral conjunctiva image synthesis under limited-data conditions. The proposed framework provides a promising approach for generating realistic synthetic conjunctiva images with controllable clinical and demographic characteristics.

## 1. Introduction

Deep learning has achieved significant advances in medical image analysis, demonstrating strong performance in tasks such as disease detection, segmentation, and computer-aided diagnosis [1]. Palpebral conjunctiva imaging has attracted interest as a non-invasive approach for hemoglobin assessment and anemia screening because of the highly vascularized nature of the conjunctiva and its relatively limited influence from melanin pigmentation [2,3]. Previous studies have reported associations between conjunctival appearance and hemoglobin levels using smartphone-acquired RGB images [2,4]. Prior efforts to address limited dataset size have primarily relied on traditional geometric augmentation techniques, such as rotation, flipping, and translation [5,6,7,8]. However, publicly available conjunctiva imaging datasets remain limited in size and demographic diversity [4], highlighting the need for approaches that can generate additional synthetic images while preserving clinically relevant characteristics.

Generative models provide an alternative means of producing synthetic medical images by learning the distribution of real images and generating new samples with similar characteristics [9]. Generative Adversarial Networks (GANs) have been widely investigated for synthetic image generation, including DCGAN, conditional GANs, and StyleGAN-based architectures [10,11]. StyleGAN has demonstrated the ability to generate realistic retinal fundus images, with ophthalmologists achieving near-random accuracy when distinguishing synthetic images from real images [12]. StyleGAN2-ADA further addressed limited-data settings through adaptive augmentation, demonstrating improved image quality, reduced overfitting, and enhanced training stability across medical imaging applications [13]. Conditional GANs have also enabled controllable image synthesis by incorporating predefined clinical or demographic attributes into the generation process [11]. Nevertheless, GAN-based approaches may experience training instability, mode collapse, and difficulty in capturing the full variability of complex image distributions [14,15].

Generative modeling for conjunctiva imaging remains relatively unexplored. To the best of our knowledge, only one previous study investigated conjunctiva image generation using DCGAN [16]. However, that work primarily focused on the downstream classification task and provided limited evaluation of the generated images and their generative characteristics. Thus, there remains a gap in the systematic development and evaluation of generative frameworks for realistic and controllable palpebral conjunctiva image synthesis, particularly under limited-data conditions.

Diffusion models have recently emerged as an alternative generative framework capable of producing realistic and diverse images while supporting flexible conditioning mechanisms [17]. Latent Diffusion Models (LDMs) perform the diffusion process in a lower-dimensional latent space, providing a computationally efficient framework for high-quality image generation [18]. Their potential for controllable medical image synthesis has been demonstrated across several imaging domains. For example, ref. [19] used LDMs to generate high-resolution 3D brain MRI images conditioned on demographic and clinical variables, demonstrating realistic and diverse image generation. Diffusion-based approaches have also been explored for dermatology imaging, where generation was controlled using disease and skin-tone attributes [20], and for pediatric chest X-ray synthesis, where image-to-image LDMs were conditioned on disease-specific information [21]. These studies demonstrate the potential of diffusion models to incorporate clinically relevant conditioning information while generating realistic synthetic medical images.

Despite these developments, diffusion-based generation has not yet been investigated for palpebral conjunctiva image synthesis. This gap provides the rationale for exploring a conditioning-guided LDM for this imaging domain. In particular, the ability to incorporate continuous clinical information together with categorical conditioning variables provides an opportunity to generate synthetic conjunctiva images with explicitly controlled characteristics. Accordingly, this study hypothesized that a conditioning-guided LDM could generate realistic and controllable palpebral conjunctiva images under limited-data conditions while achieving improved image realism and conditioning consistency compared with representative GAN-based approaches.

The objective of this study was therefore to develop and evaluate a conditioning-guided LDM based on Stable Diffusion v1.5 for controllable synthetic palpebral conjunctiva image generation using an image-to-image generation pipeline. The generation process was conditioned on continuous hemoglobin values together with gender and country information. The proposed framework was evaluated through complementary analyses of image realism, conditioning consistency, frequency-domain characteristics, zero-shot cross-population evaluation, blinded clinical assessment, and computational efficiency, with comparisons against cDCGAN and StyleGAN2-ADA. Through this evaluation, the study provides a comprehensive assessment of the realism, controllability, structural characteristics, and clinical plausibility of diffusion-generated palpebral conjunctiva images under limited-data conditions.

## 2. Materials and Methods

**Figure 1:**
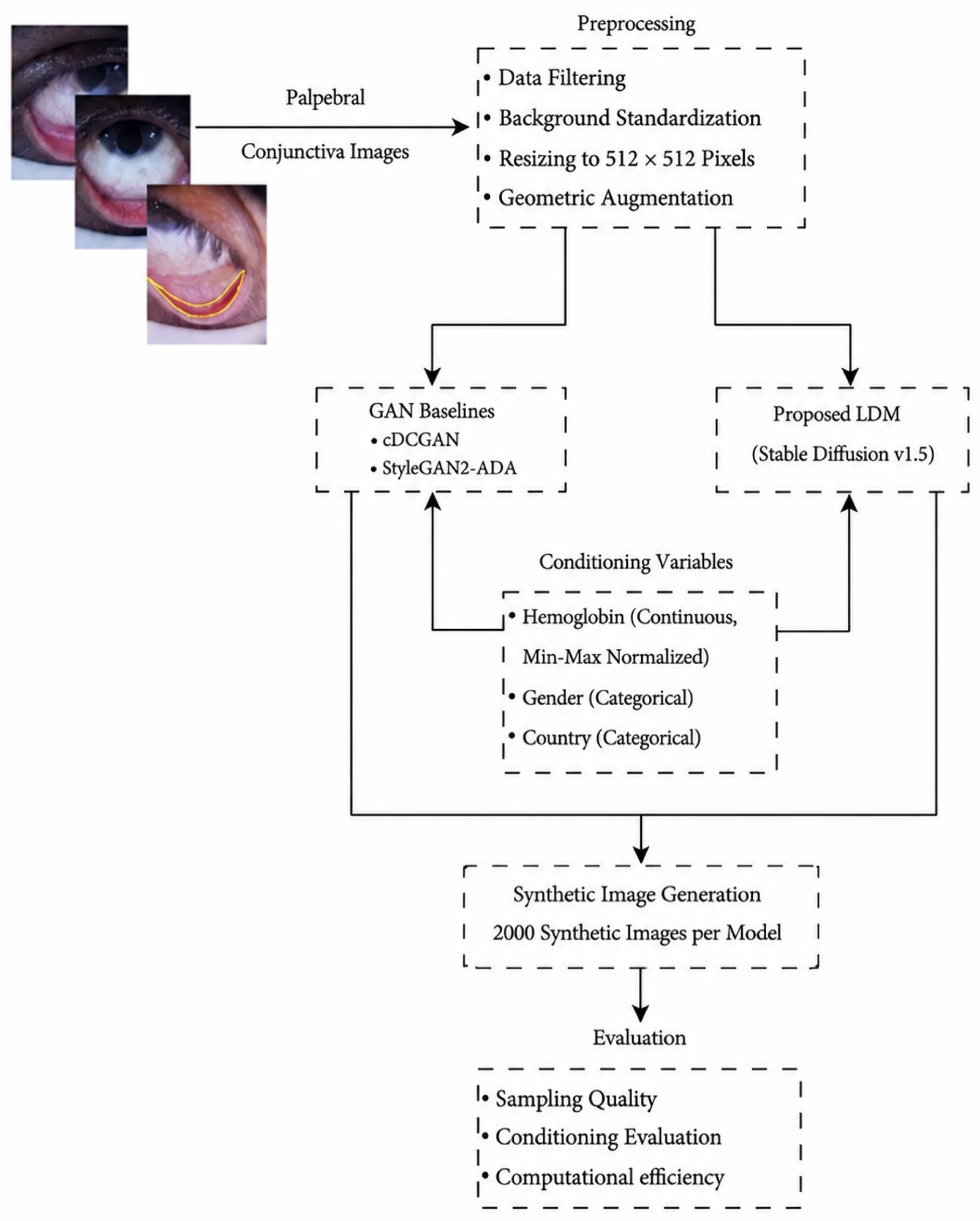
Overview of the proposed methodology.

This study was designed as an experimental artificial intelligence study for the development and evaluation of a conditioning-guided latent diffusion framework for synthetic palpebral conjunctiva image generation under limited-data conditions. The study included dataset preparation, model development, conditional image generation, and comprehensive quantitative and qualitative evaluation of the generated images. This section describes the dataset, image preprocessing procedures, the proposed generative frameworks, and the synthetic image generation process. Figure 1 presents an overview of the proposed methodology, which consists of three main stages. First, the EDA dataset undergoes data filtering, background standardization, image resizing, and geometric augmentation to prepare the training images. Next, the preprocessed images are used to train three generative models: a conditional Deep Convolutional GAN (cDCGAN), StyleGAN2-ADA, and the proposed Latent Diffusion Model (LDM) based on Stable Diffusion v1.5, all conditioned on hemoglobin level, gender, and country of origin. Finally, the trained generative models were used to generate synthetic palpebral conjunctiva images, which were subsequently evaluated through complementary analyses of image realism, diversity, conditioning consistency, frequency-domain characteristics, zero-shot cross-population evaluation, blinded clinical assessment, and computational efficiency.

### 2.1. Dataset and Image Preprocessing

The proposed generative models were developed using the EYES-DEFY-ANEMIA (EDA) dataset [4], a publicly available dataset for non-invasive anemia assessment from palpebral conjunctiva images. The original dataset contains 218 subjects from Italy and India. One subject with missing hemoglobin annotation was excluded, resulting in a final dataset of 217 subjects.

Table 1 summarizes the demographic and clinical characteristics of the dataset. The dataset consists of 122 Italian and 95 Indian subjects, including 131 males and 86 females. Laboratory-measured hemoglobin (Hb) values range from 7.0 to 17.4 g/dL, with a mean value of 12.80±2.36 g/dL.

**Figure 2:**
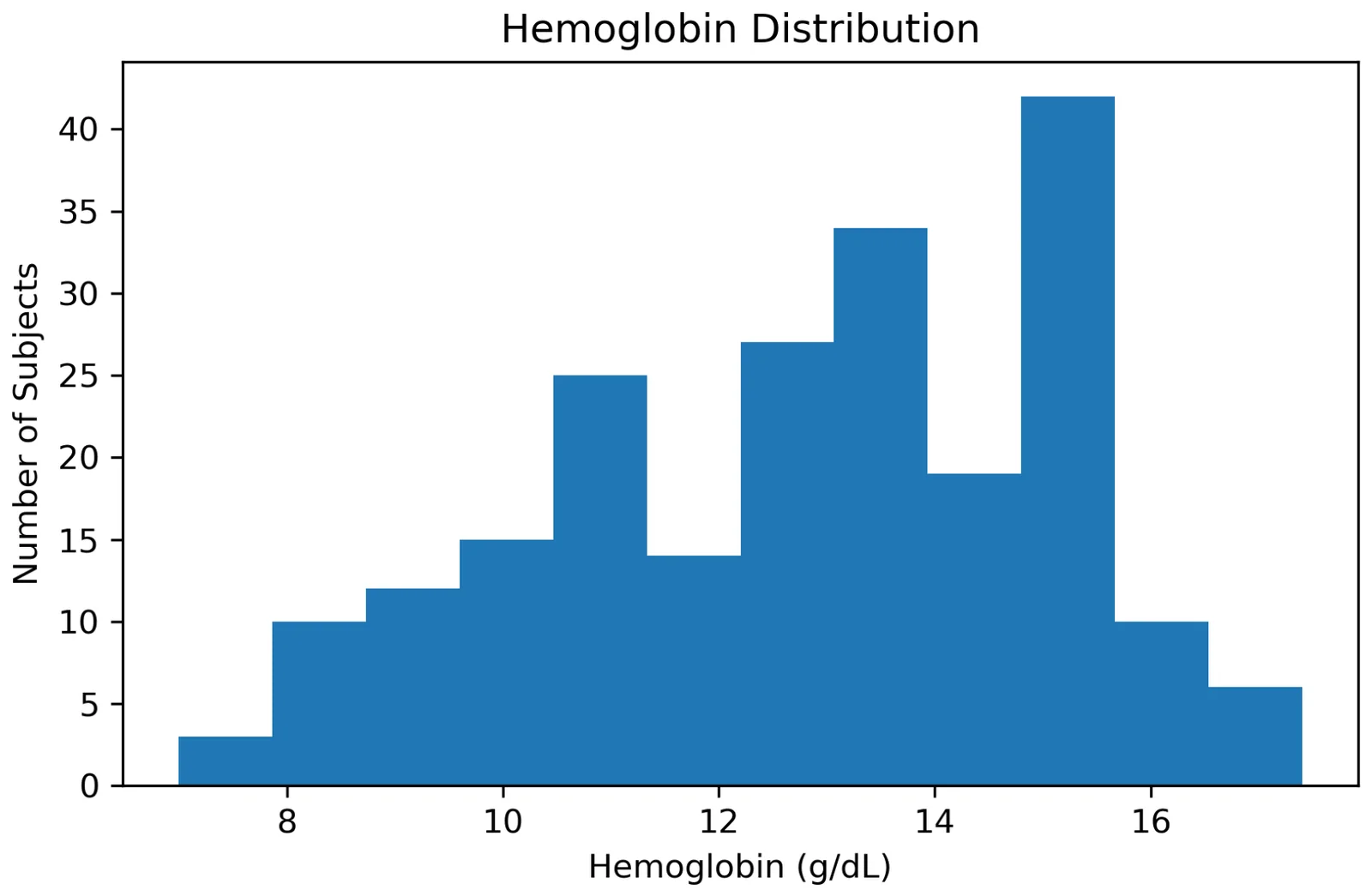
Distribution of hemoglobin values in the EDA dataset.

The distribution of hemoglobin values in the dataset is presented in Figure 2. The dataset covers a broad range of hemoglobin levels, providing sufficient variability for continuous conditioning during synthetic image generation.

**Figure 3:**
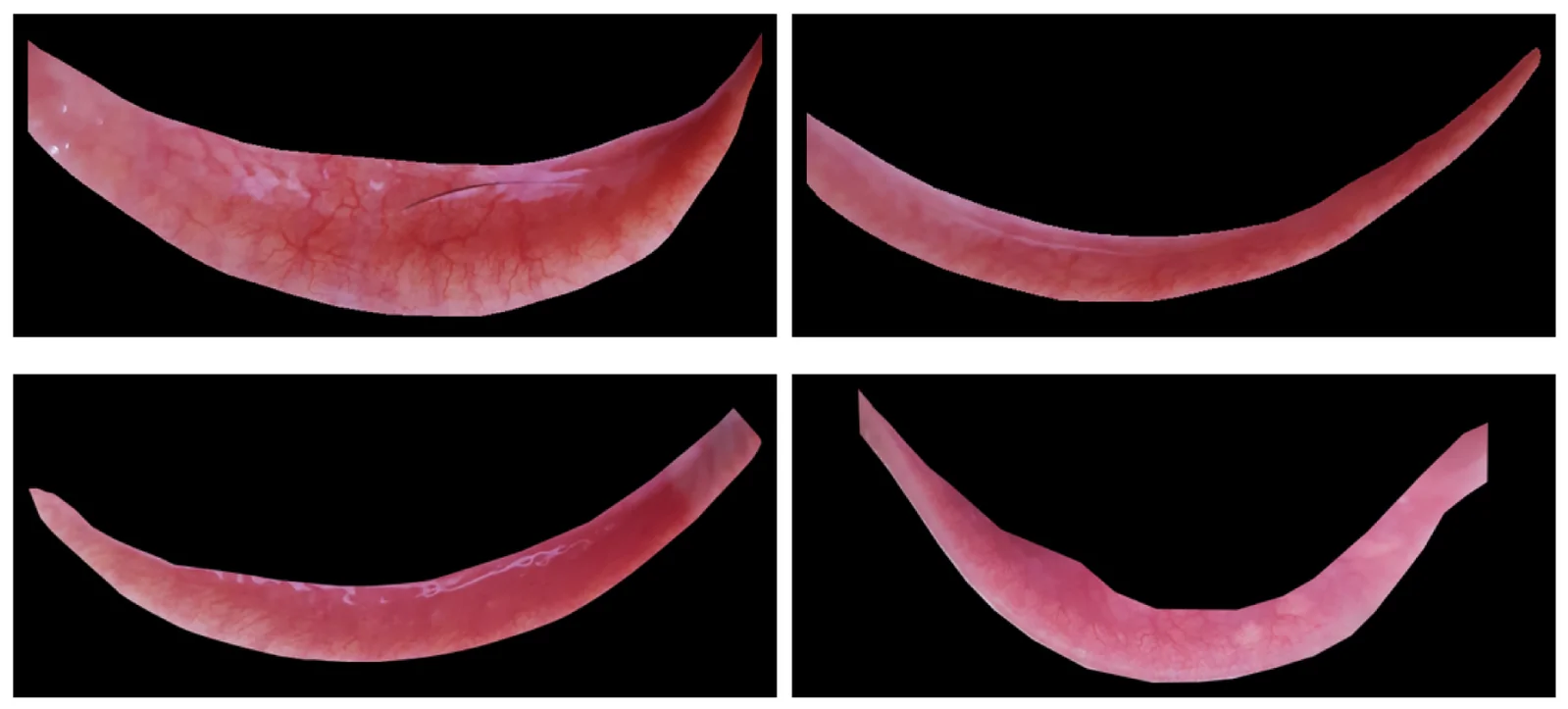
Sample palpebral conjunctiva images used as inputs for training the proposed generative models.

The EDA dataset provides manually segmented palpebral conjunctiva images for each subject in addition to the original eye images. Since the objective of this study is to generate palpebral conjunctiva images, the pre-segmented palpebral conjunctiva images provided by the dataset authors were directly used as the input images for training the proposed generative models. Representative examples of these images are shown in Figure 3.

Prior to model training, all images were resized to 512×512 pixels while preserving their three-channel RGB representation to provide a consistent input resolution across the evaluated generative frameworks. Background standardization was applied to ensure a uniform image representation across samples. To increase the number of available training samples under the limited-data setting, geometric-only augmentation techniques were applied to the training images, expanding the training set from 217 to 651 images. Geometric transformations were used to increase spatial variation while preserving the underlying appearance of the palpebral conjunctiva. These augmented images were subsequently used to train the proposed LDM, cDCGAN, and StyleGAN2-ADA.

### 2.2. Generative Frameworks

The proposed study employs three conditional generative models for synthetic palpebral conjunctiva image generation. A conditional Deep Convolutional Generative Adversarial Network (cDCGAN) and StyleGAN2-ADA were adopted as comparative GAN-based baselines, while a Latent Diffusion Model (LDM) based on Stable Diffusion v1.5 was employed as the proposed model. The cDCGAN was selected as a conventional conditional GAN baseline because it provides a relatively simple convolutional adversarial framework for controllable image synthesis, while StyleGAN2-ADA was included as an advanced GAN-based baseline because its adaptive discriminator augmentation mechanism was specifically proposed to stabilize GAN training in limited-data regimes [22]. Together, these baselines provide a comparison between a conventional conditional GAN, a modern style-based GAN with adaptive augmentation, and the proposed diffusion-based framework. All three models utilized the same conditioning variables, namely hemoglobin level, gender, and country of origin, enabling comparison under identical clinical and demographic settings. These models were selected to compare conventional and advanced GAN-based approaches with the proposed diffusion-based approach under the same conditioning strategy. The experiments were conducted on a high-performance computing (HPC) cluster equipped with an NVIDIA A100 PCIe GPU (NVIDIA Corporation, Santa Clara, CA, USA) with 40 GB VRAM. This computing environment was used for training the evaluated generative models, synthetic image generation, and all quantitative evaluations.

Before describing the individual generative models, the conditioning variables common to all models are first introduced. These variables provide the clinical and demographic information used to guide the image generation process, ensuring a consistent conditioning strategy across all models.

#### 2.2.1. Conditioning Variables

All generative models were conditioned on hemoglobin level, gender, and country of origin. Hemoglobin values were normalized using min–max normalization and provided as continuous conditioning inputs, whereas gender and country of origin were represented as categorical conditioning variables. For categorical conditioning, an “unknown” state was implemented for gender and country to allow the corresponding conditioning information to be omitted when unavailable, rather than imposing a specific demographic condition.

Hemoglobin level was selected as the primary conditioning variable because it is the primary clinical variable associated with each image and enables the generation of synthetic conjunctiva images across different hemoglobin levels. Gender was included to account for demographic variations in hemoglobin distribution. Country was incorporated as a conditioning variable to preserve the distributional characteristics of the EDA dataset during image generation, given the documented differences in demographic and clinical characteristics between the Indian and Italian subsets [4]. Together, these conditioning variables enable controllable image synthesis while preserving the clinical and demographic characteristics represented in the original dataset.

#### 2.2.2. cDCGAN-Based Generative Baseline

To provide a comparative generative baseline, a conditional Deep Convolutional Generative Adversarial Network (cDCGAN) was adopted for synthetic palpebral conjunctiva image generation. The framework follows the standard noise-to-image generation paradigm, where synthetic conjunctiva images are generated directly from random latent vectors conditioned on clinical and demographic attributes. The conditioning variables described in Section 2.2.1 were encoded using a conditioning encoder and incorporated into both the generator and discriminator networks to guide image synthesis according to the specified clinical and demographic attributes. This architecture provides a conventional conditional GAN reference for comparison with the more advanced StyleGAN2-ADA and the proposed diffusion-based framework under the same conditioning strategy.

##### Generator Architecture

The generator was implemented as a conditional Deep Convolutional GAN architecture. Random latent vectors sampled from a Gaussian distribution were concatenated with the conditioning representation generated by the conditioning encoder. The resulting feature representation was progressively transformed through multiple transposed convolution layers with batch normalization and ReLU activation functions to generate 512×512 RGB palpebral conjunctiva images.

##### Discriminator Architecture

The discriminator was implemented using a conditional PatchGAN architecture. The conditioning representation was projected into a spatial feature map and incorporated into the discriminator input. The discriminator consisted of multiple convolutional layers with batch normalization and LeakyReLU activations, producing patch-level real-versus-synthetic predictions.

The discriminator was trained to distinguish real conjunctiva images from generated images while simultaneously considering the associated conditioning variables. This conditional discrimination strategy guides the generator to produce images that are both visually realistic and consistent with the specified clinical and demographic attributes.

##### Training Procedure

The generator, discriminator, and conditioning encoder were jointly optimized using the adversarial training paradigm. Binary Cross-Entropy (BCE) loss was employed as the adversarial objective, where the discriminator learned to distinguish real and synthetic images, while the generator optimized to generate images that could successfully fool the discriminator.

Model optimization was performed using the Adam optimizer with cosine annealing learning-rate scheduling. Gradient clipping was additionally applied during training to improve optimization stability.

##### Hyperparameter Selection

Because the optimal training configuration could not be assumed a priori for the limited conjunctiva dataset, hyperparameter ablation experiments were conducted to investigate the effects of training duration, learning rate, latent-space dimensionality, and generator feature-map size on image generation quality. The effects of individual hyperparameters were first evaluated independently, followed by combination experiments to identify a suitable joint configuration. Image quality was quantitatively assessed using the Fréchet Inception Distance (FID).

As shown in Table 2, substantial performance variations were observed across different configurations. The hyperparameter ablation experiments identified a latent dimension of 64 as the best-performing latent representation among the tested latent dimensions. The subsequent combination experiments showed that the configuration consisting of a latent dimension of 64, a generator capacity of 64 feature maps, and a learning rate of $2\times 10^{-4}$ achieved the lowest FID among the tested combinations, with an FID of 102.57.

#### 2.2.3. StyleGAN2-ADA-Based Generative Baseline

To provide a more robust GAN-based comparison, StyleGAN2-ADA [22] was adopted as a comparative baseline for synthetic palpebral conjunctiva image generation. The model employs a style-based generator architecture together with adaptive discriminator augmentation (ADA), which dynamically adjusts augmentation strength during training according to the discriminator’s behavior. The conditioning variables described in Section 2.2.1 were encoded into a single conditioning vector and incorporated into the mapping network, enabling a consistent comparison with both the cDCGAN baseline and the proposed LDM under identical clinical and demographic conditioning.

##### Generator and Discriminator Architecture

The official StyleGAN2-ADA architecture was adopted while preserving its standard style-based generation mechanism, including the mapping network, weight demodulation, noise injection layers, and adaptive augmentation pipeline. The conditioning input was adapted to accommodate the clinical and demographic variables used in this study: hemoglobin level, gender, and country of origin were concatenated into a single conditioning vector and processed by the mapping network in place of the categorical class labels used in the original implementation. The generator and discriminator otherwise followed the standard StyleGAN2-ADA configuration for 512×512 image synthesis.

##### Training Procedure

To mitigate the limited size of the training dataset, StyleGAN2-ADA was initialized via transfer learning from publicly available FFHQ-512 pre-trained weights rather than random initialization, thereby leveraging a large-scale pre-trained generative prior, following a strategy analogous to the pre-trained backbone employed by the proposed LDM. The model was subsequently fine-tuned on the training set for a training budget of 1000 kimg, using the Adam optimizer with adaptive discriminator augmentation enabled throughout training.

##### Hyperparameter Selection

Because the default StyleGAN2-ADA configuration was developed for a different image domain and dataset scale, targeted hyperparameter ablation experiments were conducted to determine whether adjustments to the learning rate and latent-space dimensionality would improve generation quality on the conjunctiva dataset. Image quality was quantitatively assessed using the Fréchet Inception Distance (FID). As shown in Table 3, reducing the learning rate from the default value of $2.5\times 10^{-3}$ to $1\times 10^{-3}$ yielded the largest improvement in image quality, achieving the lowest FID score among all tested configurations, while reducing the latent dimension from 512 to 256 provided a smaller improvement over the default configuration. A combination experiment incorporating both modifications did not further improve the FID compared with the learning-rate-only configuration. Consequently, the configuration with a learning rate of $1\times 10^{-3}$ and a latent dimension of 512 was adopted for all subsequent experiments and comparisons with the proposed latent diffusion framework.

#### 2.2.4. Latent Diffusion-Based Image Synthesis Framework

Following the Stable Diffusion v1.5 framework, which is based on the Latent Diffusion Model (LDM) architecture proposed by [18], a conditioning-guided image-to-image latent diffusion framework is employed for controllable palpebral conjunctiva image synthesis. Unlike conventional diffusion models operating directly in pixel space, the adopted framework performs the diffusion process in a compressed latent space learned by a pre-trained Variational Autoencoder (VAE), reducing computational complexity while preserving image quality.

The conditioning variables described in Section 2.2.1 were encoded using a conditioning encoder and projected to match the cross-attention dimension of the Stable Diffusion UNet. The resulting embeddings were injected into the denoising UNet through the cross-attention mechanism, enabling controllable generation according to the specified clinical and demographic attributes.

For synthetic image generation, the proposed framework adopted a conditioning-guided image-to-image latent diffusion strategy to preserve anatomical consistency while introducing controlled clinical and demographic variations. Unlike conventional diffusion models that generate images from pure random noise, the proposed framework utilized real palpebral conjunctiva images as structural initialization. Each input image was first encoded into a latent representation using the frozen VAE encoder, after which controlled perturbation was introduced by partially noising the latent representation.

The perturbed latent representation was subsequently denoised using DDIM sampling [23] with 35 inference steps, during which hemoglobin level, gender, and country information were incorporated through the cross-attention mechanism to guide the generation process toward the desired clinical and demographic attributes. This formulation enables controllable image generation according to the specified conditioning variables while preserving clinically relevant anatomical structures.

##### Training Procedure

During training, RGB palpebral conjunctiva images were encoded into latent representations using the pre-trained VAE encoder. The VAE remained frozen throughout training to preserve the latent representations learned during large-scale pre-training and to reduce the risk of overfitting on the relatively small conjunctiva dataset. Consequently, only the denoising UNet and the conditioning modules were optimized.

Random Gaussian noise was progressively added to the latent representations according to the diffusion noise schedule. The denoising UNet was trained to predict the injected noise while simultaneously incorporating the conditioning information through the cross-attention mechanism. Model optimization was performed using the AdamW optimizer with an initial learning rate of $1\times 10^{-5}$ and cosine annealing scheduling. The model was trained for 120 epochs.

##### Hyperparameter Selection

To identify the optimal image generation configuration, hyperparameter ablation experiments were conducted to evaluate the influence of image-to-image noise strength and DDIM sampling steps on generation quality. Six candidate noise-strength ranges spanning [0.18,1.00] and three DDIM sampling-step settings were investigated. Image quality was quantitatively assessed using the Fréchet Inception Distance (FID), while structural preservation for the noise-strength analysis was additionally evaluated using the Structural Similarity Index (SSIM) [24] computed between each generated image and its corresponding image-to-image source image.

For the image-to-image noise-strength analysis, increasing the noise strength consistently degraded both image realism and anatomical structure preservation, as summarized in Table 4. Specifically, the FID increased from 21.49±0.63 at [0.18,0.30] to 119.36±3.17 at [0.85,1.00], while the corresponding SSIM decreased from 0.967±0.001 to 0.286±0.024. These results indicate that noise-strength values approaching or exceeding 0.70 substantially compromise anatomical fidelity.

**Figure 4:**
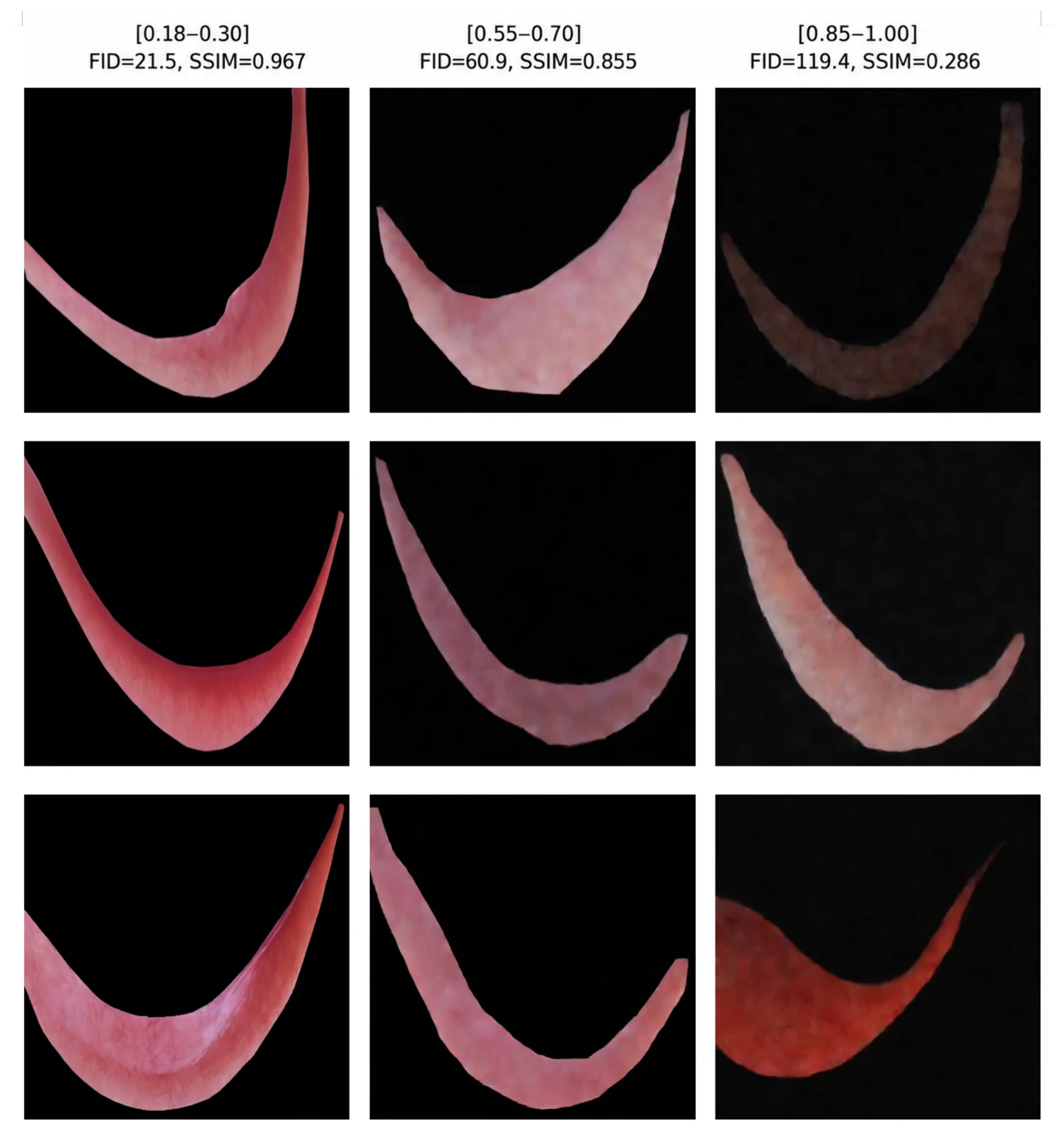
Representative synthetic palpebral conjunctiva images generated under different image-to-image noise-strength ranges. Increasing the noise strength progressively reduces the preservation of fine anatomical details and overall anatomical fidelity, consistent with the quantitative deterioration observed in FID and SSIM. The selected range of [0.18,0.30] provides the best balance between image realism and preservation of clinically relevant anatomical characteristics, whereas higher noise levels (≥0.70) lead to a noticeable loss of fine anatomical details.

Qualitative examples generated under the evaluated noise-strength ranges are presented in Figure 4, visually demonstrating the progressive loss of fine anatomical details as the noise strength increases.

For the DDIM sampling-step analysis, three inference-step settings (35, 50, and 75) were evaluated using FID, as summarized in Table 5. Among the tested configurations, 35 sampling steps achieved the lowest FID score and were therefore selected for all subsequent experiments.

Accordingly, a noise-strength range of [0.18,0.30] together with 35 DDIM sampling steps was adopted for all subsequent experiments and synthetic image generation. This choice is consistent with the implementation reported by Zhu et al. [25], who used a noise strength of 0.2 for the medical image dataset PathMNIST, compared with 0.5 for the natural-image datasets evaluated in the same study.

### 2.3. Synthetic Image Generation

Using the optimized configurations identified through the hyperparameter ablation experiments, synthetic palpebral conjunctiva images were generated using the proposed LDM together with the cDCGAN and StyleGAN2-ADA baselines. For each generative framework, 2000 synthetic images were produced. During image generation, continuous hemoglobin values together with gender and country information were used as conditioning variables. The generated images were subsequently evaluated using quantitative and qualitative sampling quality, conditioning consistency, frequency-domain, statistical, and clinical analyses.

## 3. Results

This section presents the qualitative and quantitative evaluation results of the proposed diffusion-based framework. The experimental results are organized into six main parts: sampling quality, conditioning evaluation, frequency-domain analysis, cross-population generalizability, clinical evaluation, and computational performance.

### 3.1. Sampling Quality

**Figure 5:**
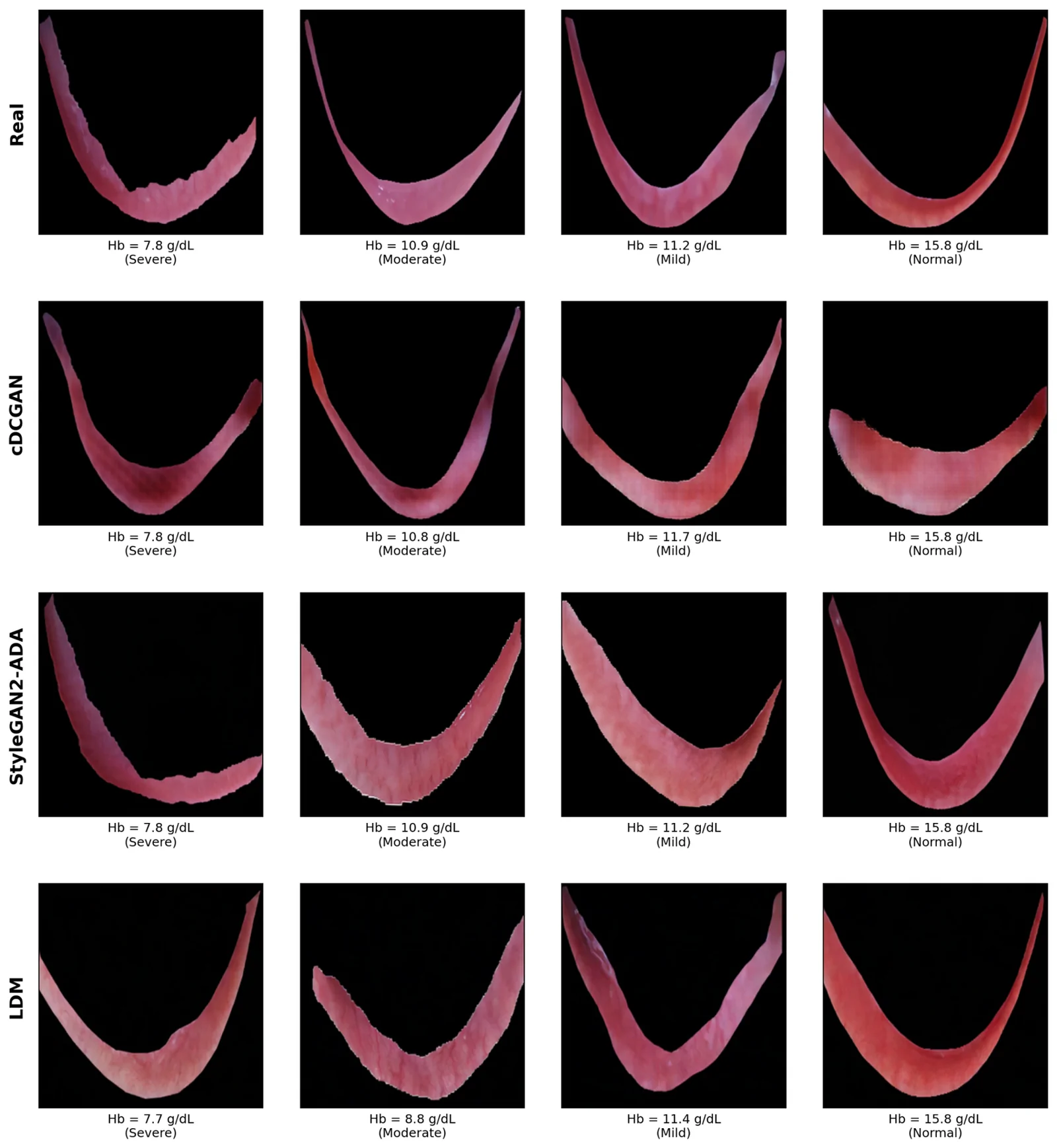
Real and synthetic palpebral conjunctiva images generated using cDCGAN, StyleGAN2-ADA, and the proposed LDM.

Qualitative comparisons between real images, the cDCGAN baseline, StyleGAN2-ADA, and the proposed LDM are presented in Figure 5. Compared with the GAN-based baselines, the proposed LDM generated higher-quality palpebral conjunctiva images with sharper anatomical structures and more realistic texture patterns.

A quantitative evaluation of the generated images was further performed using multiple realism and diversity metrics. Fréchet Inception Distance (FID) [26] and Kernel Inception Distance (KID) [27] were used to evaluate the realism of the generated samples by measuring the distributional similarity between synthetic and real palpebral conjunctiva images, where lower values indicate better generation quality.

To assess generation diversity, the Multi-Scale Structural Similarity Metric (MS-SSIM) and 4-G-R-SSIM [28,29] were additionally computed, where lower similarity values generally indicate higher diversity among generated samples. The 4-G-R-SSIM metric was additionally used because of its improved sensitivity to structural and gradient-based image characteristics. Table 6 summarizes the quantitative evaluation results obtained for the different image generation models.

The proposed LDM achieved the lowest FID score of 15.86±0.23, substantially outperforming both cDCGAN and StyleGAN2-ADA. StyleGAN2-ADA achieved slightly lower KID, MS-SSIM, and 4-G-R-SSIM values than the proposed LDM. Overall, both the proposed LDM and StyleGAN2-ADA substantially outperformed the conventional cDCGAN baseline across all evaluation metrics.

To further assess whether these differences were statistically significant, paired Student’s *t*-tests were performed across multiple independent training seeds to compare the proposed LDM with StyleGAN2-ADA. The proposed LDM achieved a significantly lower FID (p=0.0021), indicating better image realism according to the FID metric. In contrast, no statistically significant differences were observed for KID (p=0.1059), MS-SSIM (p=0.2053), or 4-G-R-SSIM (p=0.2897), indicating comparable performance between the two models with respect to these complementary image-quality metrics.

### 3.2. Conditioning Evaluation

To quantitatively evaluate conditioning consistency, an EfficientNet-B2 regression model was trained using subject-level GroupKFold cross-validation, ensuring that no subject overlap occurred between the training and validation sets. The regression model was trained exclusively on real palpebral conjunctiva images and was subsequently applied to synthetic images generated by StyleGAN2-ADA and the proposed LDM. Given its substantially lower image realism than the other generative models, the cDCGAN baseline was excluded from the conditioning analysis. Conditioning consistency was assessed by measuring the Pearson correlation between the target conditioning hemoglobin values and the corresponding values predicted from the generated images.

**Figure 6:**
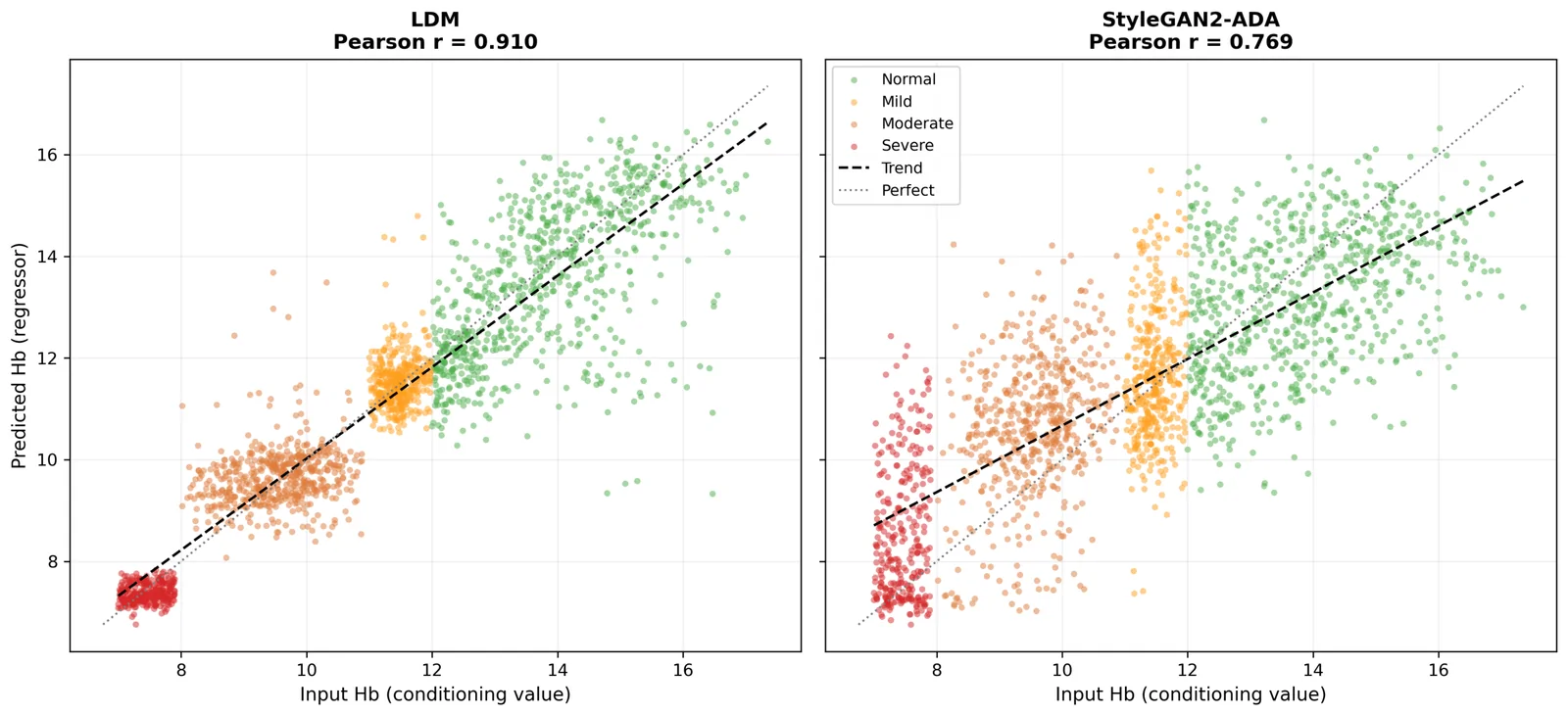
Conditioning evaluation of StyleGAN2-ADA and the proposed LDM. Scatter plots show the relationship between the target conditioning hemoglobin values and the corresponding predicted hemoglobin values obtained from the generated synthetic palpebral conjunctiva images. The proposed LDM achieved a stronger conditioning correlation than StyleGAN2-ADA.

The quantitative conditioning results are summarized in Table 7, while the corresponding scatter plots are presented in Figure 6. The proposed LDM exhibited a stronger correlation between the target and predicted hemoglobin values, achieving a Pearson correlation coefficient of 0.910±0.025 compared with 0.769±0.005 for StyleGAN2-ADA. Moreover, the predictions produced by the proposed LDM were distributed more closely around the identity line, indicating more accurate preservation of the specified hemoglobin conditioning values across the generated images.

A paired Student’s *t*-test performed across the cross-validation folds further confirmed that the improvement achieved by the proposed LDM was statistically significant (t=13.98, p<0.001). These results demonstrate that the proposed latent diffusion framework preserved the conditioning information more consistently than StyleGAN2-ADA.

**Figure 7:**
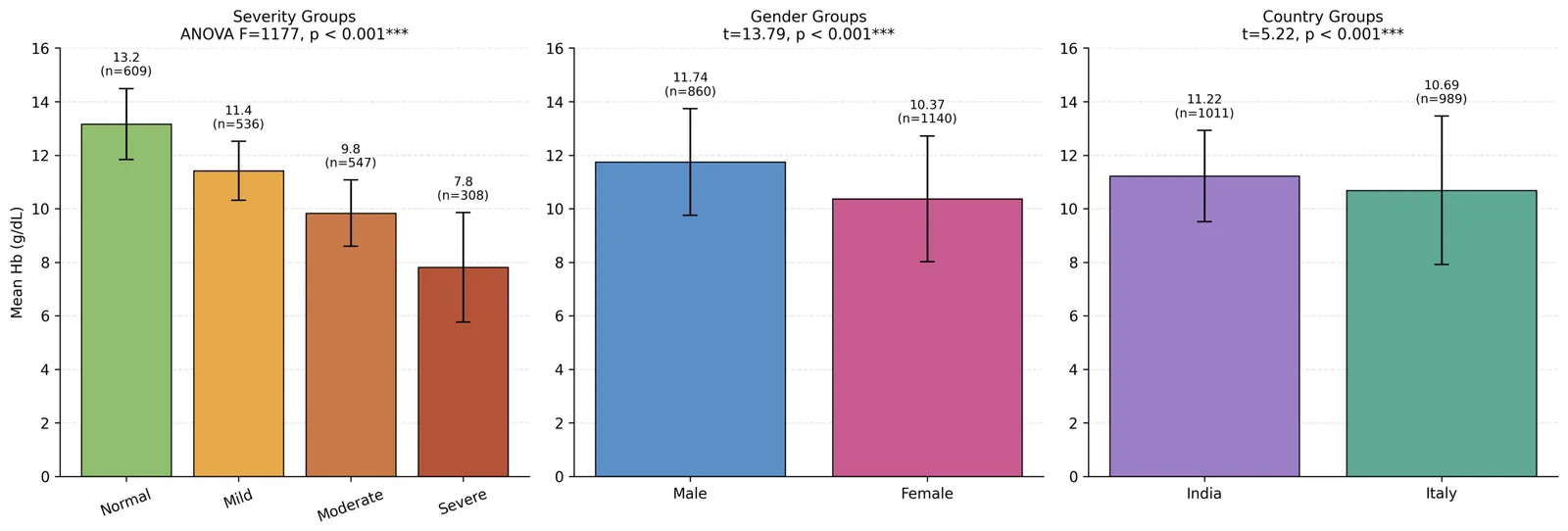
Statistical validation of the generated synthetic palpebral conjunctiva images across clinical and demographic subgroups. The figure presents subgroup-wise predicted hemoglobin distributions according to anemia severity, gender, and country. Significant differences were observed across all evaluated subgroups (p<0.001). Error bars represent standard deviations. *** indicates p<0.001.

To further evaluate conditioning consistency, statistical analyses were conducted across the clinical and demographic subgroups, as shown in Figure 7. A one-way ANOVA revealed significant differences among the four anemia severity groups (F=1177.4, p<0.001), with mean predicted hemoglobin values of 13.2, 11.4, 9.8, and 7.8 g/dL for the Normal, Mild, Moderate, and Severe groups, respectively. Independent *t*-tests further showed significant differences between the generated male and female images (t=13.79, p<0.001) and between the generated images from the two countries (t=5.22, p<0.001). These results indicate that the generated images exhibited statistically significant differences across the specified clinical and demographic conditioning groups.

### 3.3. Frequency-Domain Analysis

To further evaluate the spectral consistency of the generated conjunctiva images, a frequency-domain analysis was performed using Fourier spectrum representations on matched real and synthetic image pairs. This analysis complements conventional image quality evaluation by providing additional insight into the spectral characteristics of the generated images [30]. For each synthetic image, a corresponding real image was selected by matching the gender and country attributes while restricting the hemoglobin difference to |ΔHb|≤0.5 g/dL.

**Figure 8:**
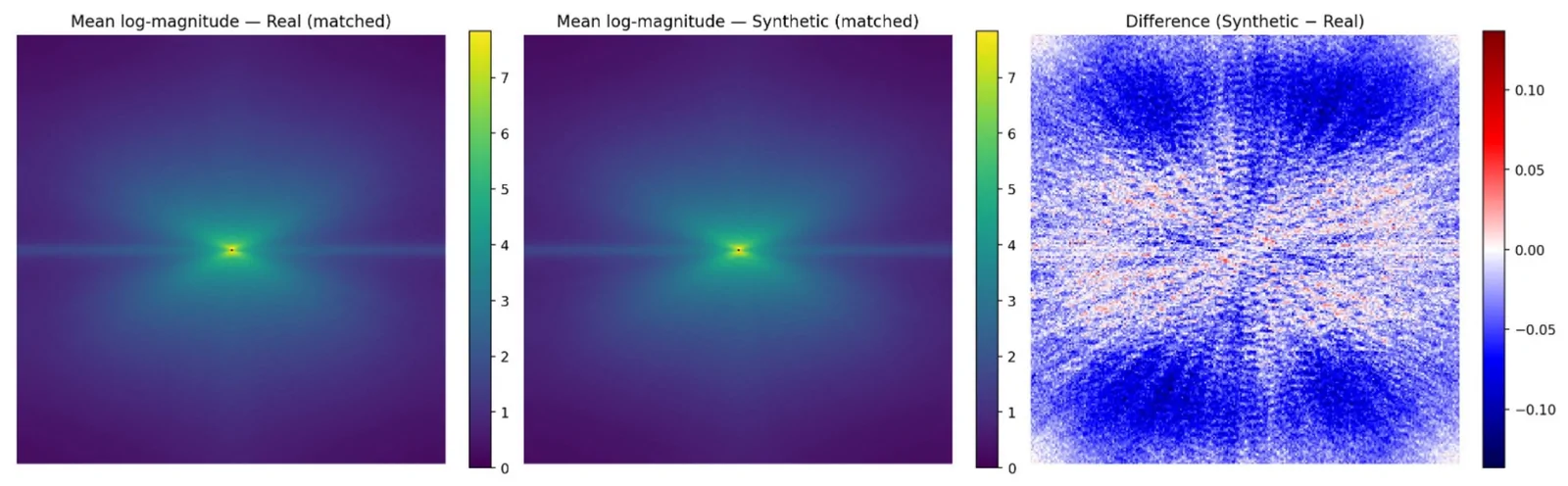
Frequency-domain comparison between matched real and synthetic conjunctiva images. The figure presents the mean Fourier log-magnitude spectra for the matched real and synthetic images together with the spectral difference map (Synthetic − Real). The generated images exhibited highly similar global frequency characteristics with only subtle localized spectral deviations.

Figure 8 presents the mean Fourier log-magnitude spectra for the matched real and synthetic images, together with the spectral difference map. The generated images exhibited highly similar global frequency distributions compared with the real conjunctiva images, indicating strong spectral consistency between the two domains. Although subtle localized spectral deviations were observed in the difference map, the overall Fourier characteristics remained closely aligned.

**Figure 9:**
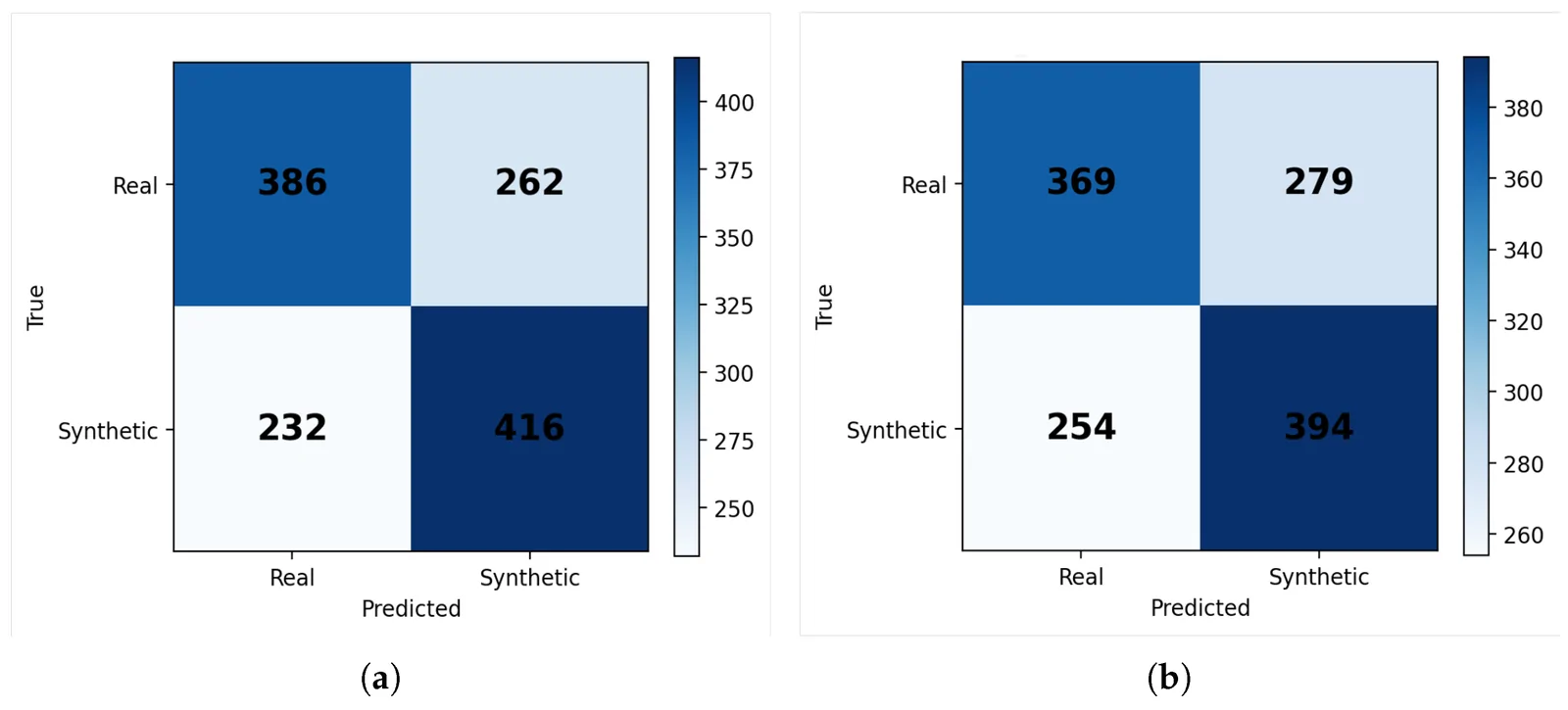
Confusion matrices for Fourier-domain real-versus-synthetic classification using Logistic Regression (accuracy = 0.619) and SVM-RBF (accuracy = 0.589). Both classifiers achieved only moderate discrimination performance, indicating strong spectral similarity between the generated synthetic conjunctiva images and the corresponding real images under matched demographic and hemoglobin conditions. (**a**) Logistic Regression; (**b**) SVM-RBF.

To further assess distinguishability in the frequency domain, multiple machine learning classifiers were trained to differentiate real and synthetic images using Fourier-based features [31]. As shown in Figure 9, both evaluated classifiers achieved only moderate discrimination performance, suggesting that the generated synthetic conjunctiva images exhibit high spectral similarity to the corresponding real images under matched demographic and hemoglobin conditions.

### 3.4. Zero-Shot Cross-Population Evaluation

Although the proposed framework was developed for controllable palpebral conjunctiva image generation using adult data, its transferability under a cross-population domain shift was further investigated using the publicly available CP-AnemiC dataset [32]. CP-AnemiC consists of pediatric palpebral conjunctiva images collected for anemia assessment in children aged 6–59 months. Despite the differences in population characteristics, imaging conditions, and acquisition protocol, the dataset provides the closest publicly available benchmark for evaluating the robustness of the proposed image generation framework under a clinically relevant domain shift.

For the zero-shot cross-population evaluation, the model trained on the EYES-DEFY-ANEMIA dataset was directly applied to CP-AnemiC images without any retraining or domain adaptation. The available gender information in CP-AnemiC was retained as a conditioning variable, whereas country was set to the predefined “unknown” state because the external population was not represented by the country categories used during training. This allowed the model to utilize the available demographic information without imposing an unsupported country condition. Continuous hemoglobin conditioning values were sampled within the hemoglobin range represented in the pediatric dataset using the same conditioning strategy employed during training. The quantitative results of the zero-shot cross-population evaluation are presented in Table 8.

**Figure 10:**
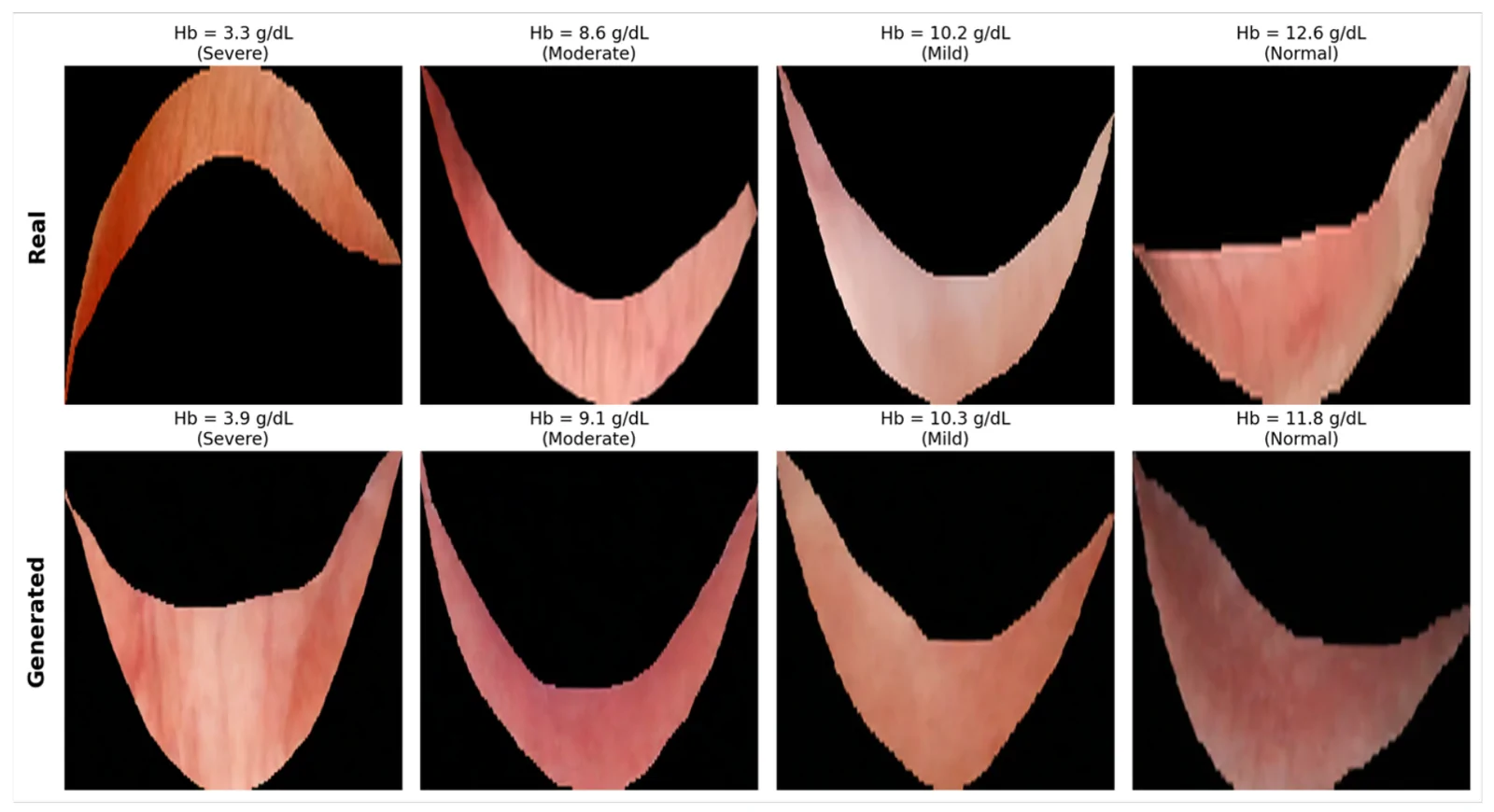
Representative zero-shot image generation results on the CP-AnemiC pediatric dataset. The first row shows real pediatric palpebral conjunctiva images, while the second row presents synthetic images generated by directly applying the LDM trained on the EYES-DEFY-ANEMIA dataset without target-domain adaptation. Across the evaluated hemoglobin levels, the generated images preserve anatomically plausible conjunctival structures despite the substantial population shift from adults to pediatric subjects.

As expected, the zero-shot setting produced a higher FID because the model was trained exclusively on adult palpebral conjunctiva images and evaluated on an independent pediatric dataset acquired under different imaging conditions. Nevertheless, the generated images preserved generation diversity comparable to that observed in the real pediatric images, as indicated by the nearly identical MS-SSIM and 4-G-R-SSIM values. Representative examples are presented in Figure 10, where the generated images maintain anatomically plausible conjunctival structures across the evaluated hemoglobin levels despite the substantial population shift. These findings suggest that the proposed latent diffusion framework retains reasonable structural consistency under substantial population shifts while highlighting the remaining challenges of transferring generative medical image models across distinct clinical domains without target-domain adaptation.

### 3.5. Clinical Evaluation

To further assess the clinical plausibility of the generated images, a blinded reader study was conducted involving two independent clinical experts. Each clinical expert independently evaluated a randomized subset of 20 palpebral conjunctiva images, comprising 10 real and 10 synthetic images, without being informed of their origin. For each image, the clinical experts determined whether the image was real or synthetic, assigned an overall realism score, and evaluated the physiological plausibility of the visible capillary pattern.

Table 9 summarizes the results of the blinded clinical evaluation. The discrimination accuracies achieved by the two clinical experts were 50% and 60%, indicating that neither clinical expert could reliably distinguish synthetic images from real images. Furthermore, Mann–Whitney U tests revealed no statistically significant differences between real and synthetic images in either realism scores or capillary pattern quality ratings, with all comparisons yielding p>0.05. These findings support the clinical plausibility and anatomical realism of the images generated by the proposed framework.

### 3.6. Computational Performance

To provide a practical comparison of the computational efficiency of the evaluated generative models, the training time and inference time were measured under the same hardware environment. Table 10 summarizes the computational cost of cDCGAN, StyleGAN2-ADA, and the proposed LDM, while Table 11 lists the hardware and software environment used throughout the experiments.

Although the proposed LDM required longer inference time than GAN-based models because of the iterative denoising process, its training time was substantially lower than StyleGAN2-ADA while producing higher-quality synthetic images. The experiments were conducted on an NVIDIA A100 GPU under the software environment summarized in Table 11.

## 4. Discussion

This study investigated the use of a conditioning-guided Latent Diffusion Model (LDM) for controllable synthesis of palpebral conjunctiva images under limited-data conditions. Unlike conventional GAN-based approaches, the proposed framework simultaneously incorporated continuous hemoglobin values together with demographic conditioning variables through a cross-attention mechanism, enabling clinically guided image generation while preserving anatomically meaningful conjunctival structures. Comprehensive evaluation was performed using image realism, diversity, conditioning consistency, frequency-domain analysis, zero-shot cross-population evaluation, blinded clinical assessment, and computational efficiency.

The qualitative and quantitative evaluations consistently demonstrated that the proposed LDM generated higher-quality conjunctiva images than the conventional cDCGAN baseline. Among all evaluated generative models, the proposed framework achieved the lowest FID score, indicating the highest distributional similarity between the generated and real conjunctiva images. Although StyleGAN2-ADA achieved slightly lower KID, MS-SSIM, and 4-G-R-SSIM values, these differences were not statistically significant. Statistical analysis further confirmed that only the improvement in FID reached statistical significance, suggesting that the primary advantage of the proposed LDM lies in producing images that more closely follow the distribution of real conjunctiva images while maintaining diversity comparable to that of StyleGAN2-ADA. These findings are consistent with previous diffusion-based medical imaging studies, which reported improved generation realism, stability, and diversity compared with GAN-based approaches under limited-data settings [19,20]. These observations are also consistent with previous studies reporting that diffusion models generally produce more realistic medical images than GAN-based approaches because image synthesis is formulated as an iterative denoising process rather than adversarial optimization [17,33].

The superior realism observed in the proposed framework may be partly related to two complementary design choices. First, operating in the latent space reduces the complexity of the diffusion process while leveraging representations learned by the pre-trained variational autoencoder. Second, the adopted image-to-image generation strategy uses real conjunctival images as structural guidance during the diffusion process, while controlled noise injection and conditional generation introduce variations according to the specified conditioning variables. This combination may support anatomically plausible synthesis under limited-data conditions while maintaining controlled variability in the generated images.

The conditioning experiments further evaluated the consistency between the specified conditioning variables and the generated images. Compared with StyleGAN2-ADA, the proposed LDM achieved a substantially stronger correlation between the conditioning hemoglobin values and the corresponding predicted hemoglobin values, indicating closer agreement between the specified and predicted hemoglobin levels. The scatter plots further showed that the generated samples were distributed considerably closer to the identity line, suggesting that the generated images were generally consistent with the intended hemoglobin conditioning. In addition, statistically significant differences were observed across the specified anemia severity, gender, and country groups. These findings indicate that the generated images exhibited systematic differences across the conditioning groups, consistent with the conditioning information provided to the model. Overall, the results suggest that the proposed cross-attention conditioning mechanism was able to incorporate both continuous and categorical conditioning information during image generation. These findings are consistent with previous conditional diffusion studies in medical imaging, where diffusion-based conditioning mechanisms have been used to generate images corresponding to continuous or categorical clinical variables [19].

Frequency-domain analysis provided additional evidence that the proposed framework preserved image characteristics beyond the spatial domain. The mean Fourier spectra of the generated and real conjunctiva images exhibited highly similar global frequency distributions, while the Fourier-based classifiers achieved only moderate discrimination performance. Together, these findings suggest that the generated images contain only subtle spectral discrepancies relative to real conjunctiva images. Since frequency-domain artifacts are often associated with generative models, the observed spectral similarity further supports the realism of the proposed diffusion-based synthesis framework.

The cross-population evaluation further demonstrated encouraging generalization capability. Without any retraining or domain adaptation, the model trained exclusively on the adult EYES-DEFY-ANEMIA dataset was directly applied to the pediatric CP-AnemiC dataset. As expected, image realism decreased under this zero-shot setting because of the considerable differences in population characteristics and acquisition conditions between the two datasets. Nevertheless, the generated pediatric images preserved generation diversity comparable to that of the real pediatric images, while qualitative inspection showed anatomically plausible conjunctival structures across the evaluated hemoglobin levels. These findings suggest that the proposed framework learned anatomical characteristics that remain partially transferable across clinically distinct populations, although additional domain adaptation would likely be required for optimal performance in new acquisition settings.

The blinded clinical evaluation further supported the practical realism of the generated images. Neither clinical expert was able to reliably distinguish synthetic images from real images, with discrimination accuracies remaining close to chance level. Moreover, no statistically significant differences were observed between real and synthetic images with respect to overall realism or physiological plausibility of the capillary patterns. These observations indicate that the proposed framework generated anatomically plausible conjunctiva images that were visually consistent with real clinical images from the perspective of experienced clinical experts.

From a computational perspective, the proposed framework also demonstrated a practical balance between image quality and computational cost. Although inference required more time than the GAN-based models because of the iterative denoising process, the total training time remained substantially lower than that of StyleGAN2-ADA while achieving superior image realism. Overall, the proposed LDM provided a favorable trade-off between computational cost and image quality.

The potential clinical implications of realistic and controllable synthetic conjunctiva images extend beyond image generation itself. Such images may provide a useful supplementary source of data for future hemoglobin prediction studies, particularly in settings where real conjunctiva images are limited or unevenly distributed across hemoglobin levels and demographic groups. The ability to control the conditioning variables may also facilitate the development of more diverse datasets for investigating model robustness across different patient populations and acquisition settings. Furthermore, synthetic image generation may support future multicenter studies by providing an additional means of exploring population and acquisition variability when sufficiently large real datasets are not readily available. These potential applications remain to be investigated in future downstream and multicenter studies.

Several limitations should nevertheless be acknowledged. First, the proposed framework was trained and evaluated using a relatively limited number of conjunctiva images collected from only two countries. Although encouraging cross-population results were obtained using CP-AnemiC, additional validation on larger multi-center datasets acquired under different clinical conditions would further establish the generalizability of the proposed framework. Second, conditioning evaluation relied on a regression model trained on real conjunctiva images rather than direct physiological measurements of the generated samples. Finally, while the reader study provided preliminary evidence of clinical realism, larger multi-reader evaluations involving ophthalmologists and hematologists would provide a more comprehensive assessment of clinical utility.

Future work will focus on expanding the proposed framework using larger multi-center conjunctiva datasets to further improve its robustness across diverse imaging conditions and patient populations. Although encouraging zero-shot cross-population results were observed on the CP-AnemiC pediatric dataset, future research will investigate domain adaptation techniques to better bridge the distributional differences between adult and pediatric conjunctiva images. In addition, incorporating additional clinically relevant conditioning variables and evaluating the proposed framework through larger multi-reader clinical studies may further improve both the controllability and the clinical validation of the generated images. Finally, the proposed conditioning-guided latent diffusion framework may be extended to other ophthalmic image synthesis and medical image augmentation applications where annotated datasets remain limited.

Overall, the experimental results demonstrate that the proposed conditioning-guided latent diffusion framework provides an effective approach for controllable palpebral conjunctiva image synthesis under limited-data conditions. The framework generated anatomically realistic images with superior realism, strong conditioning consistency, evidence of structural consistency across populations, and strong clinical plausibility, while providing a practical trade-off between image quality and computational cost.

## 5. Conclusions

In this study, a conditioning-guided latent diffusion framework was proposed for controllable synthesis of palpebral conjunctiva images under limited-data conditions. The proposed framework incorporated continuous hemoglobin values together with demographic conditioning variables through a cross-attention mechanism, enabling clinically guided image generation while preserving anatomically meaningful conjunctival structures.

Experimental results demonstrated that the proposed framework generated realistic conjunctiva images with the lowest FID among the evaluated generative models while maintaining image diversity comparable to StyleGAN2-ADA. In addition, the proposed conditioning strategy showed strong consistency with the target hemoglobin values and systematic differences across the demographic conditioning groups, whereas frequency-domain analysis, zero-shot cross-population evaluation, and blinded clinical assessment further supported the realism, structural consistency, and clinical plausibility of the generated images.

Overall, the proposed conditioning-guided latent diffusion framework provides an effective approach for controllable conjunctiva image synthesis and offers a promising strategy for synthetic data generation. Its potential utility for dataset augmentation and for improving conjunctiva-based hemoglobin prediction or anemia-screening performance remains to be evaluated in future downstream studies.

## Figures and Tables

**Table 1:** Demographic and clinical characteristics of the EDA dataset used in this study.

Characteristic	Value
Total Subjects	217
Country Distribution	
Italy	122 (56.2%)
India	95 (43.8%)
Gender Distribution	
Male	131 (60.4%)
Female	86 (39.6%)
Hemoglobin Range	7.0–17.4 g/dL
Mean Hemoglobin ± SD	12.80±2.36 g/dL

**Table 2:** Summary of cDCGAN hyperparameter ablation and combination experiments. Lower FID (↓) indicates better image realism.

Experiment	Tested Settings	FID ↓
Epochs	80 120 180 260 300 340 400 500	166.35 ± 31.69 227.41 ± 26.89 145.42 ± 15.79 136.19 ± 3.41 129.02 ± 16.21 161.99 ± 6.79 179.49 ± 41.08 154.94 ± 26.73
Learning Rate	$2\times 10^{-4}$ $1\times 10^{-4}$ $5\times 10^{-5}$ $4\times 10^{-4}$	211.56 166.90 296.55 194.25
Latent Dimension	32 64 128 256	118.78 102.57 274.77 167.71
Generator Feature Maps	32 64 128	145.07 343.67 188.82
Combination Experiments	z64 + ngf32 + lr = $2\times 10^{-4}$ z64 + ngf32 + lr = $1\times 10^{-4}$ z64 + ngf64 + lr = $2\times 10^{-4}$ z64 + ngf64 + lr = $1\times 10^{-4}$ z64 + ngf128 + lr = $2\times 10^{-4}$ z64 + ngf128 + lr = $1\times 10^{-4}$	303.97 204.05 102.57 168.86 113.80 213.90

**Table 3:** Summary of StyleGAN2-ADA hyperparameter ablation and combination experiments. Lower FID (↓) indicates better image realism.

Experiment	Tested Settings	FID ↓
Learning Rate	$2.5\times 10^{-3}$ $1\times 10^{-3}$	30.15 22.35
Latent Dimension	512 256	30.15 28.84
Combination Experiment	lr $1\times 10^{-3}$ + latent dim 256	23.11

**Table 4:** Effect of image-to-image noise strength on image realism and anatomical structure preservation in the proposed latent diffusion framework. Lower FID (↓) and higher SSIM (↑) indicate better image realism and structural preservation, respectively.

Noise Strength Range	FID ↓	SSIM ↑
[0.18,0.30]	21.49±0.63	0.967±0.001
[0.25,0.45]	32.65±0.66	0.953±0.001
[0.35,0.55]	40.90±0.56	0.934±0.002
[0.55,0.70]	60.93±1.16	0.855±0.008
[0.70,0.85]	81.37±1.35	0.622±0.024
[0.85,1.00]	119.36±3.17	0.286±0.024

**Table 5:** Effect of DDIM sampling steps on image generation quality. Lower FID (↓) indicates better image realism and distributional similarity to real images.

DDIM Sampling Steps	FID ↓
35	21.55±0.62
50	22.87±0.69
75	22.67±0.73

**Table 6:** Quantitative evaluation of the synthetic images on the EYES-DEFY-ANEMIA dataset. Fréchet Inception Distance (FID) and Kernel Inception Distance (KID) were used to evaluate image realism, while MS-SSIM and 4-G-R-SSIM were used to assess generation diversity. Lower values (↓) are preferred for all metrics.

Model	FID ↓	KID ↓	MS-SSIM ↓	4-G-R-SSIM ↓
Real Images	13.91	0.001 ± 0.006	0.594 ± 0.100	0.622 ± 0.061
cDCGAN	102.57	0.056 ± 0.021	0.550 ± 0.043	0.592 ± 0.021
StyleGAN2-ADA	23.03 ± 0.59	0.0071 ± 0.0019	0.5065 ± 0.0016	0.5495 ± 0.0071
LDM	15.86 ± 0.23	0.0096 ± 0.0007	0.5088 ± 0.0015	0.5560 ± 0.0009

**Table 7:** Conditioning consistency evaluation of the generated synthetic images. Pearson correlation coefficients were computed between the target conditioning hemoglobin values and the corresponding predicted hemoglobin values. Higher values (↑) indicate higher correlation.

Model	Pearson (*r*) ↑
StyleGAN2-ADA	0.769±0.005
LDM	0.910±0.025

**Table 8:** Zero-shot cross-population evaluation on the CP-AnemiC pediatric dataset. FID measures image realism, while MS-SSIM and 4-G-R-SSIM evaluate generation diversity. Lower values (↓) are preferred for all metrics.

Model	FID ↓	MS-SSIM ↓	4-G-R-SSIM ↓
Real Pediatric Images	28.66±0.94	0.398±0.060	0.497±0.035
Zero-shot (Adult → Pediatric)	69.99±0.0001	0.401±0.006	0.496±0.003

**Table 9:** Results of the blinded clinical evaluation conducted by two independent clinical experts.

Clinical Expert	Discrimination Accuracy	Realism (*p*)	Capillary Quality (*p*)
Expert 1	50%	0.69	0.19
Expert 2	60%	0.61	0.78

**Table 10:** Training and inference time comparison of the evaluated generative models.

Model	Training Time	Inference Time/Image
cDCGAN	∼0.75 h (300 epochs)	0.613±0.012 ms
StyleGAN2-ADA	17.39±0.05 h (1000 kimg)	6.22±0.12 ms
LDM	3.12±0.04 h (120 epochs)	0.390±0.001 s

**Table 11:** Hardware and software environment used for training and evaluation.

Component	Specification
GPU	NVIDIA A100 PCIe (40 GB VRAM)
Python	3.12.9
PyTorch	2.5.1
CUDA	12.4
torchmetrics	1.8.2
diffusers	0.36.0
piq	0.8.0

## Data Availability

The dataset analyzed during the current study is publicly available from its original source, while the synthetic dataset generated during this study is available from the corresponding author upon reasonable request.
